# Physiology-based regularization of the electrocardiographic inverse problem

**DOI:** 10.1007/s11517-016-1595-5

**Published:** 2016-11-21

**Authors:** Matthijs J. M. Cluitmans, Michael Clerx, Nele Vandersickel, Ralf L. M. Peeters, Paul G. A. Volders, Ronald L. Westra

**Affiliations:** 1 Department of Data Science and Knowledge Engineering and CARIM School for Cardiovascular Diseases, Maastricht University, Maastricht, The Netherlands; 2 Department of Physics and Astronomy, Ghent University, Ghent, Belgium; 3 Department of Data Science and Knowledge Engineering, Maastricht University, Maastricht, The Netherlands; 4 CARIM School for Cardiovascular Diseases, Maastricht University, Maastricht, The Netherlands

**Keywords:** Electrocardiographic imaging, Electrocardiography, Cardiology

## Abstract

The inverse problem of electrocardiography aims at noninvasively reconstructing electrical activity of the heart from recorded body-surface electrocardiograms. A crucial step is regularization, which deals with ill-posedness of the problem by imposing constraints on the possible solutions. We developed a regularization method that includes electrophysiological input. Body-surface potentials are recorded and a computed tomography scan is performed to obtain the torso–heart geometry. Propagating waveforms originating from several positions at the heart are simulated and used to generate a set of basis vectors representing spatial distributions of potentials on the heart surface. The real heart-surface potentials are then reconstructed from the recorded body-surface potentials by finding a sparse representation in terms of this basis. This method, which we named ‘physiology-based regularization’ (PBR), was compared to traditional Tikhonov regularization and validated using in vivo recordings in dogs. PBR recovered details of heart-surface electrograms that were lost with traditional regularization, attained higher correlation coefficients and led to improved estimation of recovery times. The best results were obtained by including approximate knowledge about the beat origin in the PBR basis.

**Electronic supplementary material:**

The online version of this article (doi:10.1007/s11517-016-1595-5) contains supplementary material, which is available to authorized users.

## Introduction

Cardiac arrhythmias are among the leading causes of death worldwide. The 12-lead electrocardiogram (ECG) is a well-established, patient-friendly, quick, reproducible and cheap tool to determine normal cardiac activation and recovery, to diagnose cardiac arrhythmias, altered activation, ischemia, infarction, primary electrical abnormalities of the heart, structural disease, metabolic disorders, electrolyte imbalance and other conditions. It reflects the attenuated and dispersed result of propagated electrical activity and recovery in the heart on the body surface.

However, it lacks the capacity to directly assess electrical activity at the level of the heart muscle at high resolution. Electrocardiographic imaging (ECGI) aims at noninvasively reconstructing the electrical activity of the heart, based on body-surface potential measurements and a patient-specific torso–heart geometry [5, 18, 21, 22, 28]. This is achieved by solving what is known as the inverse problem of electrocardiography. In the last decades, much progress has been made in ECGI and clinical applications are published with increasing frequency, yet the accuracy of the reconstructed electrical heart activity is still suboptimal. This is partly due to the ill-posedness of the inverse problem: Small variations (noise) in the input data will yield unique but unrealistic variations in the reconstructions [5]. To cope with this problem, *regularization* is applied, i.e., additional knowledge is incorporated in the form of constraints on the possible solutions to attain more realistic results. Such constraints are often based on physical or mathematical properties of the problem [15, 19], but the use of electrophysiological properties has also been proposed [3, 10, 12, 13, 30].

In a previous study [3], we have shown that it may be beneficial to also include *simulated* electrophysiological input in the reconstruction process, using a method we call *physiology-based regularization* (PBR). In this method, propagating waveforms originating from several positions on the heart are simulated and used to generate a set of basis vectors representing spatial distributions of potentials on the heart surface. The real heart-surface potentials are then reconstructed from the recorded body-surface potentials as a sparse linear combination of these ‘building blocks’ on the heart surface. In other words, this new method decomposes the simulated heart-surface potential patterns into basis vectors which span the space of heart-surface potentials and then solves the inverse problem by pursuing a sparse representation of the heart-surface potentials in terms of this basis.

In this manuscript, we provide a detailed description of PBR and use in vivo recordings to assess its performance. We investigate whether PBR can improve reconstructions of epicardial ventricular potentials, specifically with the goals of detecting the origin of ectopic beats and imaging substrates for arrhythmias.

## Methods

The potential-based formulation of the forward/inverse problems of electrocardiography is based on the assumption that there is a direct relation between potentials on a closed surface surrounding the heart and the body surface [20]. The closed surface surrounding the heart is usually taken to be the epicardium, i.e., the outer myocardial layer. The forward problem can then be defined as:

1 $$\varPhi _B (t)=A\varPhi _H (t)$$

where $\varPhi _B (t)$ are the potentials on the body surface at a specific time instant *t*, $\varPhi _H (t)$ the potentials on the heart surface and *A* is the transfer matrix that relates these vectors. The transfer matrix captures the geometry and conductivity relation between the surfaces. It is assumed that the problem is quasi-static and the torso volume is source-free, and therefore, the transfer matrix is time-independent.

The goal of the inverse problem is to find the cardiac potentials $\varPhi _H (t)$ from recorded body-surface potentials $\varPhi _B (t)$ and a patient-specific transfer matrix *A* (usually based on a computed tomography (CT) scan). However, small variations in the body-surface potentials, e.g., due to noise, will result in disproportionately large changes in the computed cardiac potentials. In other words, the computed solution of the inverse problem does not depend continuously on the data and the problem is, therefore, ill-posed. Additional constraints are needed to obtain a stable, regularized solution. For example, the well-known Tikhonov method obtains a stable solution by placing bounds on the amplitude of the reconstructed cardiac potentials (or derivatives thereof) with a least squares minimization at time instant *t*:

2 $$\underset{\varPhi _H (t)}{\min } \left\{ \left\| A\varPhi _H (t)-\varPhi _B (t) \right\| _2^2 + \lambda (t) \left\| R \varPhi _H (t) \right\| _2 ^2 \right\}$$

where *R* is the regularization operator (the identity matrix for zeroth-order, the gradient operator for first-order, or the Laplacian for second-order regularization). The regularization parameter $\lambda (t)$ balances the quality of fit with the amount of regularization and can be determined with methods such as the L-curve. Recent papers review these and other regularization methods [17, 20].

**Fig. 1:**
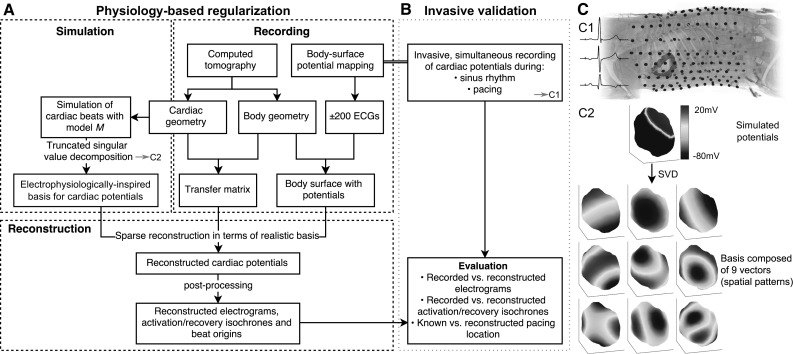
Schematic representation of physiology-based regularization (**a**) and its validation in an in vivo canine model (**b**). *c1* Body-surface electrodes (*blue*), ventricular epicardium (*green*) and epicardial electrodes (*red*) in the canine experiment. *c2* Simulated potentials on the epicardium. Taking the singular value decomposition (SVD) of many (morphologically distinct) simulated beats yields a realistic basis of electrophysiologically relevant solutions that will be used to reconstruct epicardial potentials (color figure online)

PBR is a regularization method that constrains the solutions based on patient-specific electrophysiology simulations. It is illustrated schematically in Fig. 1a. First, as in regular ECGI, body-surface potentials are recorded (Sect. 2.1) and a CT scan is performed from which both a digitization of the heart-surface geometry and the location of the body-surface electrodes are obtained (Sect. 2.2). Next, simulations of propagating action potentials (APs) originating from different points on the digitized epicardium are run (Sect. 2.3). Singular value decomposition (SVD) is applied to the combined simulated patterns of epicardial APs, and truncation is applied to arrive at a small set of basis vectors representing spatial distributions of potentials on the heart (Sect. 2.4). The real epicardial potentials are then reconstructed as sparse combinations of these vectors (Sect. 2.5).

By the nature of SVD, the resulting basis vectors span the space of simulated AP patterns on the heart surface. We assume that the simulated APs form a good surrogate for potential patterns that could be expected on a human heart, and their basis vectors thus form a suitable basis for reconstruction of true heart-surface potentials. Furthermore, by aiming for a *sparse* representation in terms of this basis, we aim to further reduce the influence of ill-posedness. It is essential to realize that, although a basis might contain only a few vectors, the space spanned by the basis contains all linear combinations of these vectors and so is still vast. Therefore, we hypothesize that a limited number of simulated beats can already provide a basis from which a huge number of electrophysiologically realistic potential patterns can be reconstructed, including many patterns not encountered in the simulations. An example of a 9-vector basis is shown in Fig. 1c.

To validate this method, we performed in vivo measurements of the epicardial potentials in three normal, anesthetized dogs, while simultaneously recording potentials at the body surface. This is illustrated in Fig. 1b, c.

### In vivo recordings

In vivo data were acquired in experiments with anesthetized dogs. This investigation conformed to the Guide for the Care and Use of Laboratory Animals published by the United States National Institutes of Health (National Institutes of Health Publication 85-23, revised 1996). Animal handling was in accordance with the European Directive for the Protection of Vertebrate Animals Used for Experimental and Other Scientific Purposes (86/609/EU) and was approved by the institutional review committee for animal studies.

In three normal anesthetized dogs, two silicone bands with 99 electrodes were implanted around the basal and mid-basal epicardium after thoracotomy [3]. Each band consisted of two rows of electrodes, and an additional electrode was placed on the LV apical epicardium. After chest closure, body-surface electrodes (184–216, depending on torso size) were attached to the chest (ActiveTwo setup, BioSemi, Amsterdam, the Netherlands). This number of body-surface electrodes is more than sufficient to obtain a good reconstruction with traditional methods [4]. Unipolar potential recordings were obtained simultaneously by the epicardial and body-surface electrodes.

Beats were recorded during normal sinus rhythm or with epicardial pacing from electrodes on the left ventricle (LV) or right ventricle (RV).

### Torso–heart geometry

A CT scan was performed and used to digitize a homogeneous geometry consisting of the body-surface electrodes and the epicardial surface. Segmentation of the surfaces from CT scans was performed manually with Seg3D [23]. The ventricular epicardium was digitized as a triangulated mesh with approximately 1700 nodes and the position of the 103 implanted electrodes was recorded. The septum was not included in this segmentation. The transfer matrix, relating the electrical activity on the cardiac surface to the body surface, was computed with methods available from the SCIrun software repository [1] and was based on a boundary elements method. In one dog, a three-dimensional digitization of the entire ventricular myocardium (including septum) was created in addition to the epicardial surface. This digitization had a much finer resolution, with grid points spaced 0.5 mm apart in the *x*, *y* and *z* directions.

### Simulation of epicardial potentials

Computational models of the AP were used to simulate waveform propagation over the digitized ventricular myocardium. To see the influence of the AP model used, we ran simulations with three different models: the neuronal FitzHugh–Nagumo model (FHN) [8], the mammalian ventricular model by Luo and Rudy (LR92) [14] and the human ventricular model by Ten-Tusscher et al. (TNNP) [24].

*Simplified simulations* For our main results, we used fast simulations on a coarse mesh representing the ventricular epicardial surface. The potential at each node in the mesh was modeled using one of the three single-cell AP models mentioned above. Currents between the nodes were introduced as $I = g \varDelta {V}$ for every two nodes connected in the triangulation. The node-to-node conductance *g* was set as $g = \bar{g}/d^2$ where *d* is the distance between the nodes and $\bar{g}$ is a parameter that can be manipulated to adjust the speed of propagation. In this simplified approach, we assumed conduction was the same in all directions and did not vary from node to node. Because PBR uses spatial but not temporal characteristics of the simulated waveforms, the exact timing of AP propagation is not important, as long as the resulting data set contains realistic spatial patterns. To achieve this, we set $\bar{g}$ to a value that reproduced the main characteristics of ventricular activation and recovery, i.e., in chronological order: a propagating front of activation, fully activated ventricles, a wave of recovery and fully recovered ventricles. These simulations were performed using Myokit, our toolkit for AP model development and simulation [2].

**Fig. 2:**
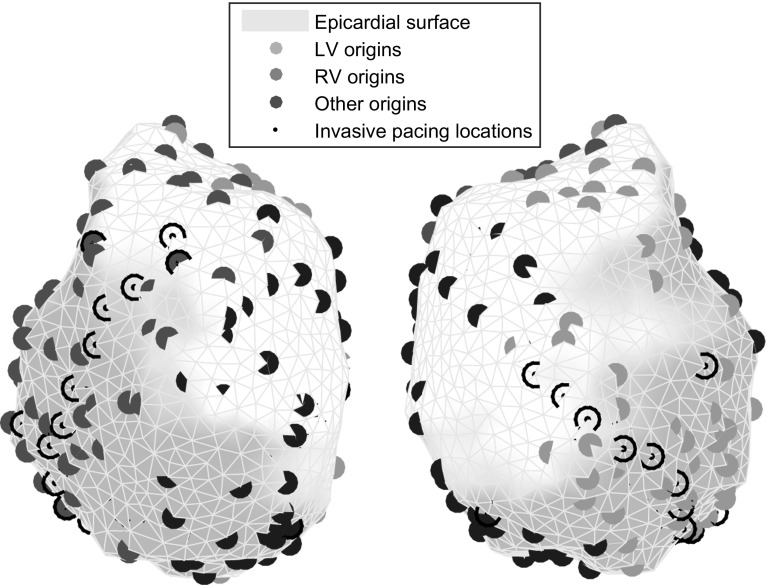
Example of the origins of 50 simulated beats on the LV (*green*), the RV (*red*) and other locations (*blue*) and invasive pacing locations (*black*). Note that the simulated origins generally do not match with the invasive pacing locations (color figure online)

*Beat origins* We performed simulations for each dog, with simulated beats originating from several, unique locations. Locations were chosen in three areas: (1) on the LV free wall, (2) on the RV free wall and (3) at the base or apex of the ventricles (where no pacing was performed during the in vivo experiment). Origin locations within these areas were chosen pseudo-randomly, and inspected visually for a roughly uniform, equidistant distribution. A single beat was simulated per origin. Where not stated otherwise, the reconstructions in this manuscript used beats from 50 origins per region. Figure 2 shows an example of the origins of simulated beats.

*Detailed simulations* The method described above is a heuristic method, which is not guaranteed to provide the same accuracy as more advanced techniques such as finite-element-based methods using the monodomain or bidomain equations (although they are based on the same physical principles). In addition, the mesh used for these simulations was rather coarse, compared to the mesh size typically used for 'whole-heart' simulations. To validate the applicability of this simplified method for PBR, we performed additional detailed simulations for one of the dogs and compared the results. In these simulations, a fine-grained, regular rectangular mesh was used, representing the entire ventricular myocardium, including the left, right and septal walls. Propagation was then simulated by solving the monodomain equations using a finite-difference approximation and assuming zero-flux boundary conditions. Again, conduction in the myocardium was assumed to be homogeneous and isotropic, as no knowledge of the fiber direction was available. In these detailed monodomain simulations, 29 origins per region were used. For accurate comparison with the simplified simulations, regions were defined as before, with all beats originating from the epicardium of the free walls (never from the endocardium or septum). The simulations were performed using a parallelized monodomain solver [29] and the TNNP model of the AP.

### Creation of a realistic basis

For each dog and model, the heart-surface APs of all simulated beats were concatenated in a single potential matrix $\varPhi _H$. The number of rows in this matrix was equal to the number of heart-surface nodes, and the number of columns equaled the number of simulated beats (typically 50) times the number of time steps per beat (typically 550).

This combined set of APs, reflecting a diversity of activation and recovery patterns, was then decomposed using singular value decomposition (SVD):

3 $$\varPhi _H = UDV^T$$

Here, the columns of *U* form a spatial basis for the simulated beats, the columns of *V* form a temporal basis and the diagonal matrix *D* represents the corresponding singular values. The entries in *D* were ordered in non-increasing order, i.e., with the largest singular values first. Each singular value can be interpreted as a weight, such that a small value indicates a small contribution of the corresponding spatial and temporal patterns to describing the overall data. A truncated spatial basis $U_k$ was created by keeping only the first *k* most influential elements. These capture the most prominent spatial patterns that build up the simulated potentials. Truncated bases can be beneficial as they leave fewer possibilities for ill-posed influences that could result in unrealistic solutions.

### Sparse reconstruction of epicardial potentials

Assuming that $U_k$ can be used as a basis for the epicardial potentials at any time *t*, there should be a vector $\beta {(t)}$ such that

4 $$\varPhi _H (t)=U_k \beta (t)$$

and our forward problem becomes

5 $$\varPhi _B (t)=A U_k \beta (t)$$

By using the truncated basis $U_k$ instead of the full basis *U*, the reconstruction is constrained to only those elements of *U* that contain the most relevant physiological information. Reconstruction of epicardial potentials in terms of this new basis can then be achieved by Lasso regularization [25]. This is a form of least squares approximation that minimizes the least squares error of the direct solution $||A U_k \beta (t)-\varPhi _B (t)||_2^2$ while at the same time requiring $||\beta ||_1$ to be smaller than a given parameter $\lambda (t)$ [25]:

6 $$\underset{\beta (t)}{\min } \left\{ \left\| A U_k \beta -\varPhi _B (t) \right\| _2 ^2 \right\} {\text { subject to }} ||\beta (t)||_1 \le \lambda (t)$$

This minimization can be solved for each time instant *t* independently. The resultant $\beta (t)$ can then be plugged into Eq. 4 to obtain the heart-surface potentials at time *t*.

$L_1$-norm penalties have previously been shown (in a different setup) to give more accurate results than commonly used $L_2$-norms [11]. Constraining the $L_1$-norm of the parameter vector $\beta (t)$ tends to produce only a few nonzero coefficients in $\beta (t)$, leading to a sparse representation of the epicardial potentials. In other words, only the most important elements of the truncated basis will be used in the reconstruction of the epicardial potentials. As this basis consists only of well-defined spatial potential patterns, we expected that this approach would reduce the influence of ill-posedness. We used MATLAB to solve this Lasso problem, choosing $\lambda (t)$ such that the mean square error was minimized [16].

We reconstructed beats with bases generated from simulated beats originating from all regions of the heart for all three AP models ($\hbox{FHN}_{\mathrm{all}}$, $\hbox{LR}92_{\mathrm{all}}$ and $\hbox{TNNPa}_{\mathrm{all}}$). For beats paced at the LV or RV, we compared these results to reconstructions based only on simulated beats originating from the appropriate region ($\hbox{FHN}_{\mathrm{spec}}$, $\hbox{LR}92_{\mathrm{spec}}$ and $\hbox{TNNPa}_{\mathrm{spec}}$). In one dog, reconstructions were performed using a generic and a region-specific basis based on detailed monodomain simulations ($\hbox{TNNPb}_{\mathrm{all}}$ and $\hbox{TNNPb}_{\mathrm{spec}}$).

### Post-processing

Activation and recovery times were determined from reconstructed electrograms with two different methods: a temporal-only method and a spatiotemporal method. The temporal-only approach defines the moment of activation as the moment of steepest voltage downslope (maximum $-d\varPhi _H/{\mathrm{d}}t$) during the QRS complex. Recovery times were defined as the moment of maximum $d\varPhi _H/{\mathrm{d}}t$ during the T wave. The spatiotemporal approach, proposed by Erem et al. [6], takes advantage of the spatial relationship between neighboring nodes and their potentials and could be better suited to estimate the activation time in noisy or fractionated electrograms. Erem et al. noted that not only the temporal signal (i.e., the local potential at a single node) changes quickly when an activation wavefront passes, but also the spatial gradient of potentials between neighboring nodes. Their approach to activation time estimation selects the moment at which the change in temporal derivative coincides with the change in spatial derivative [6]. More formally, for each epicardial node, they define the activation time $\tau$ as:

7 $$\tau = \underset{t}{\min } \left\| D \varPhi _H (t) \right\| _2 \cdot \frac{\partial \varPhi _H (t)}{\partial t}$$

where $\varPhi _H (t)$ is the potential at the epicardial node under consideration at time *t*, $D \varPhi _H(t)$ is the approximated spatial gradient, and $\partial \varPhi _H/\partial t$ the approximated temporal derivative.

### Statistical evaluation

For each epicardial electrode, Pearson’s correlation coefficient (CC) was computed between the recorded electrogram and the reconstructed electrogram at the corresponding (closest) virtual epicardial node. Linear correlation between recorded and reconstructed activation/ recovery timings was assessed by means of Pearson’s correlation coefficient R. Results were statistically compared with Wilcoxon signed-rank tests (for paired measurements) or Wilcoxon rank-sum tests (for unpaired measurements).

Note that we only compare morphology, not absolute error, as the amplitude of the reconstructed potentials depends on the amount of regularization (especially with Tikhonov zeroth-order regularization, which constrains the amplitude explicitly). Moreover, morphology is usually of much more clinical significance, as it contains information about the order and timing of activation and recovery, and can indicate local tissue abnormalities (e.g., fractionation, ST segment deviation). Therefore, we did not restrict our evaluation to comparing correlation coefficients of morphology, but also considered clinically relevant parameters such as activation timing, recovery timing and beat origin localization.

**Fig. 3:**
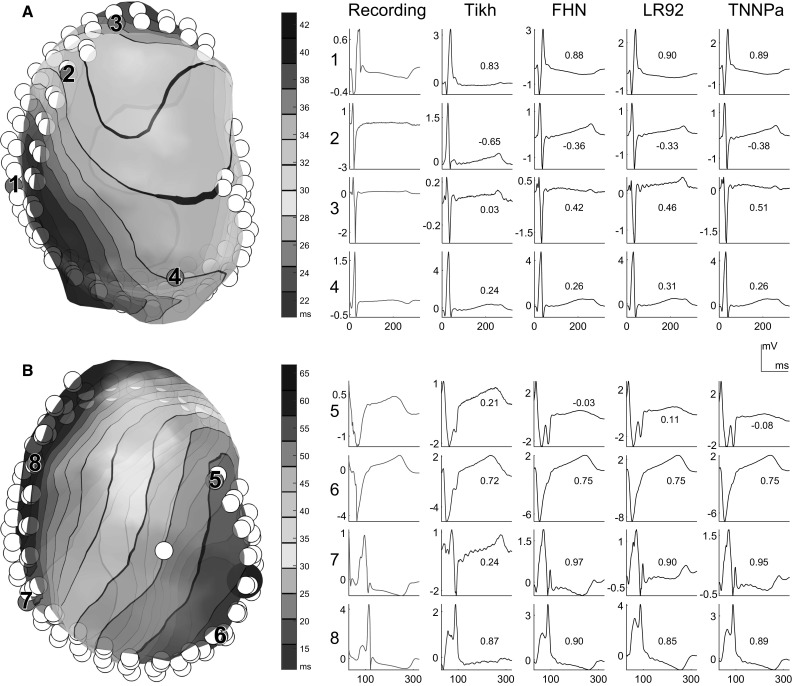
Ventricular epicardium (*left*, *colored* according to noninvasively reconstructed activation times) and recorded and reconstructed electrograms (*right*) during a sinus beat (**a**) and an LV paced beat (**b**, pacing location indicated by *blue sphere*). *White circles* represent the implanted epicardial electrodes. For selected electrodes (*purple*, numbered) the corresponding electrograms are shown: recorded (*red*), Tikhonov-reconstructed (*blue*) and electrograms reconstructed with PBR for three different basis types (FHN, LR92 or TNNPa); the *numbers* next to the electrograms indicate the correlation coefficient with the invasively recorded signal (color figure online)

## Results

Figure 3 shows examples of recorded and reconstructed electrograms for a sinus beat and a paced beat in one dog. Electrograms were reconstructed with PBR using different AP models and with traditional zeroth-order Tikhonov regularization for comparison. These examples show that, regardless of the AP model, all PBR methods were able to recover some of the electrogram characteristics that were lost with Tikhonov regularization, for example the negative deflection in electrogram 1 and 2, and the positive deflection in electrogram 7. There were no significant changes between the different models used in PBR.

**Fig. 4:**
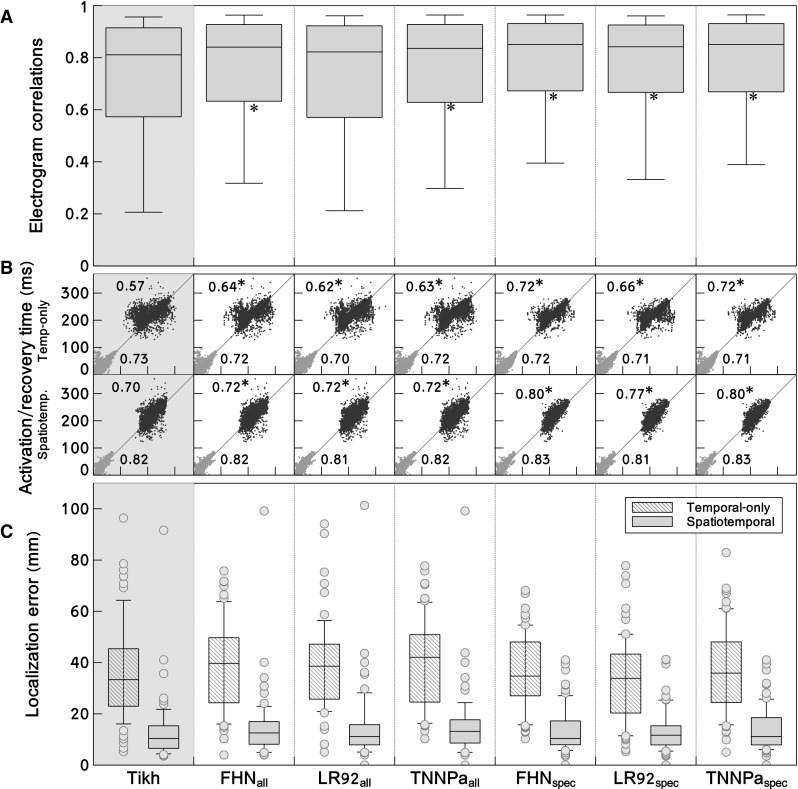
Results for the full data set. *Columns* show the result for the different reconstruction methods: traditional Tikhonov (Tikh) regularization, or regularization by a physiology-based method without ($\hbox{FHN}_{\mathrm{all}}$, $\hbox{LR}92_{\mathrm{all}}$ and $\hbox{TNNPa}_{\mathrm{all}}$) and with ($\hbox{FHN}_{\mathrm{spec}}$, $\hbox{LR}92_{\mathrm{spec}}$ and $\hbox{TNNPa}_{\mathrm{spec}}$) region-specific bases. **a** *box plots* of correlation coefficients between recorded and reconstructed electrograms. Box spans the interquartile range (IQR), i.e., the 25–75% range; median indicated by *horizontal line*; whiskers at 9–91% range. Overall, PBR improves reconstruction quality, especially using region-specific bases. **b** Activation times (*red*) and recovery times (*blue*) as reconstructed (*horizontal axes*) versus recorded (*vertical axes*). *Top row* shows these timings as directly determined from the electrograms, i.e., with temporal-only criteria. *Bottom row* shows timings as determined with a spatiotemporal method. Recovery times, especially, are improved by PBR. Additional improvement is achieved when spatiotemporal post-processing is used. **c** Localization error between detected and known origins of pacing, as determined with temporal-only methods (*hatched box plots*) and with spatiotemporal methods (*gray box plots*). A combined use of spatiotemporal post-processing and PBR gives most accurate results. An *asterisk* indicates significant improvement compared to Tikhonov results (color figure online)

In Fig. 4, quality of reconstruction is shown for all beats. Data were analyzed for all three dogs, for 88 beats in total (of which 67 were epicardially paced, while 21 followed native sinus rhythm) and for 5203 epicardial electrogram pairs (recorded vs reconstructed). Results are shown for traditional regularization with Tikhonov and for PBR using the full bases ($\hbox{FHN}_{\mathrm{all}}$, $\hbox{LR}92_{\mathrm{all}}$ and $\hbox{TNNPa}_{\mathrm{all}}$), and using region-specific bases ($\hbox{FHN}_{\mathrm{spec}}$, $\hbox{LR}92_{\mathrm{spec}}$ and $\hbox{TNNPa}_{\mathrm{spec}}$). Figure 4a shows that the correlation between recorded and reconstructed electrograms increased significantly for 5 out of 6 PBR models. This indicates that more details were recovered by the PBR methods when compared to Tikhonov regularization, as expected from Fig. 3.

In Fig. 4b, activation and recovery times are shown as determined with the temporal-only method (top row) and the spatiotemporal method (bottom row). Spatiotemporal post-processing yielded more accurate timings than the temporal-only method. Activation times determined from PBR-based electrograms were no more accurate than those determined from Tikhonov-based electrograms. However, recovery times were significantly more accurate when PBR was used, especially with region-specific bases. Combining spatiotemporal post-processing with region-specific bases gave the most accurate results (with $R=0.70$ to $R=0.80$, $p<0.05$).

Figure 4c shows that, in line with these results, localization error was improved by using spatiotemporal post-processing in all methods. Localization error did not further improve by using PBR, although using region-specific PBR did remove one outlier.

Table 1 shows the results for a subset of the data (one dog, 39 recorded beats), where we also used the bases generated with the whole-heart model (TNNPb). From this table, it can be seen that a simple model of the AP combined with a simplified propagation model gives the same quality PBR reconstruction as a detailed three-dimensional monodomain model.

**Table 1:** Effect of more complex AP model

Model	$\hbox{CC}_{\mathrm{EGM}}$	$R_{\mathrm{act}}$	$R_{\mathrm{rec}}$	LE (mm)
Tikh (reference)	0.81	0.8	0.58	39
$\hbox{FHN}_{\mathrm{all}}$	0.85*	0.77	0.62*	44*
$\hbox{LR}92_{\mathrm{all}}$	0.83*	0.77	0.59	42
$\hbox{TNNPa}_{\mathrm{all}}$	0.84*	0.77	0.60*	47*
$\mathbf{TNNPb}_{{\mathbf{all}}}$	0.84*	0.77	0.62*	45
$\hbox{FHN}_{\mathrm{spec}}$	0.87*	0.78	0.79*	39
$\hbox{LR}92_{\mathrm{spec}}$	0.87*	0.78	0.69*	39
$\hbox{TNNPa}_{\mathrm{spec}}$	0.87*	0.78	0.79*	39
$\mathbf{TNNPb}_{\mathbf{spec}}$	0.87*	0.78	0.76*	40

Median accuracy metrics in one dog (39 recorded beats), investigating the added value of a detailed whole-heart model (TNNPb)

*LE* localization error, i.e., the distance between the known pacing location and reconstructed location of earliest activation

* Statistically significant difference with Tikhonov results. Activation and recovery times (and beat origins) determined from temporal-only criteria

All previous results are based on PBR reconstructions using bases that consisted of components 2–10 of *U*, that is, the first ten components of the SVD of the simulated beats, not including the first component. The first component is a constant negative pattern, reflecting the −80 mV offset of the simulated APs. As we reconstruct electrograms (with a zero average), not APs, this offset component does not have a role for our purpose. Components 2–10 for a FHN-based set of simulated beats are shown in Fig. 1c1. In Online Fig. 1, the basis components are shown for all AP models and beat origins in one dog. Reconstruction accuracy with different components of the SVD as basis is listed in Table 2 for components 2–5, 2–10, 2–15 and 2–25 for the full data set. For most metrics, using components 2–10 gave the best result.

**Table 2:** Dependency on basis size

Basis size	Tikh	$\hbox{FHN}_{\mathrm{all}}$	$\hbox{LR}92_{\mathrm{all}}$	$\hbox{TNNPa}_{\mathrm{all}}$	$\hbox{FHN}_{\mathrm{spec}}$	$\hbox{LR}92_{\mathrm{spec}}$	$\hbox{TNNPa}_{\mathrm{spec}}$
Correlation coefficients for electrograms (CC)							
5	0.81	0.80*	0.79*	0.80*	0.83*	0.83*	0.83*
10	”	**0**.84*	**0**.**82**	**0**.84*	**0**.85*	**0**.84*	**0**.**85**
15	”	0.81	0.81	0.81	0.82	0.81	0.82*
25	”	0.74*	0.73*	0.73*	0.72*	0.74*	0.71*
Activation time correlation (R)							
5	0.73	0.68	0.66	0.68	0.65	0.65	0.64
10	”	**0**.**72**	0.70	**0**.**72**	**0**.**72**	**0**.**71**	**0**.**71**
15	”	**0**.**72**	**0**.**71**	**0**.**72**	0.70	0.67	0.69
25	”	0.62	0.57	0.62	0.51	0.58	0.49
Recovery time correlation (R)							
5	0.57	0.62*	0.60	0.62*	0.69*	0.67*	0.69*
10	”	**0**.64*	**0**.62*	**0**.63*	**0**.72*	**0**.66*	**0**.72*
15	”	0.58	0.57	0.58	0.61*	0.62*	0.61*
25	”	0.41	0.39	0.42	0.37	0.36	0.35
Localization error (mm)							
5	33	48*	45*	48*	46*	41*	45*
10	”	40	39	42*	**35**	**34**	36
15	”	**34**	**30**	**33**	**35**	36	**34**
25	”	37	45*	37	41*	38	36*

Median accuracy metrics for the full data set for different number of components of the realistic basis (basis size *k*), best results are in bold

* Statistically significant difference with Tikhonov results. Activation and recovery times (and beat origins) determined from temporal-only criteria

**Fig. 5:**
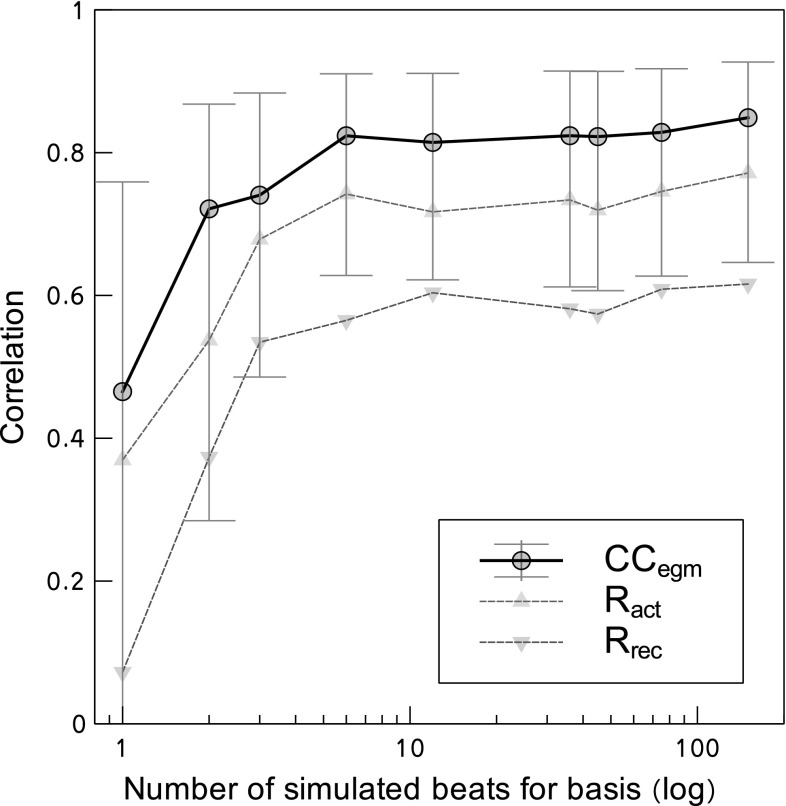
Dependency of the accuracy of PBR on the number of (unique) simulated beats that are used for the basis. Results for one dog (39 recorded beats), reconstructed with AP model $\hbox{FHN}_{\mathrm{all}}$. Correlation coefficients between recorded and reconstructed electrograms ($\hbox{CC}_{\mathrm{egm}}$) are maximal and stable when approximately 6 or more beats are simulated. Similarly, activation times ($R_{\mathrm{act}}$) and recovery times ($R_{\mathrm{rec}}$) are reconstructed with highest accuracy for 6 or more simulated beats

Not only the basis size *k* is important, but also the number of morphologically distinct beats that was used to create that basis. In Fig. 5, the dependency of PBR accuracy on the number of simulated unique beats is explored in a subset of the data (one dog, 39 recorded beats) with the $\hbox{FHN}_{\mathrm{all}}$ basis. Both correlation between recorded and reconstructed electrograms and the accuracy of activation and recovery times are maximal when data from 6 or more simulated beats are included in the basis $U_k$.

## Discussion

We have shown in an in vivo experiment that PBR increases the accuracy of reconstruction of electrograms and recovery times, compared to traditional Tikhonov regularization, and was able to recover electrogram characteristics that were lost with Tikhonov regularization. This indicates that PBR yields more information in the reconstructed electrograms than traditional methods.

### Activation and recovery times

PBR did not improve accuracy of activation times, which were already determined with reasonable accuracy using Tikhonov regularization. Activation times were most accurate when the spatiotemporal method for activation time estimation was used. In contrast, recovery times were determined more accurately with PBR than with Tikhonov regularization and therefore profited less from spatiotemporal post-processing. However, a combination of PBR with spatiotemporal post-processing yielded the highest accuracy for recovery times.

Abnormalities in recovery patterns can be an important substrate for cardiac arrhythmia and they are often difficult to diagnose from the 12-lead ECG due to its limited spatial resolution. Noninvasive imaging of recovery abnormalities could therefore greatly benefit risk assessment and further understanding of their role in arrhythmia.

### Beat origin localization

Beat origin localization is performed by finding the location with the earliest activation time. Since the accuracy of the estimated activation times did not improve significantly with PBR, beat origin locations were not significantly improved. However, some outliers were removed when region-specific bases were used. This is relevant for clinical practice, where the origin of a ventricular ectopic beat is often a target for invasive ablation therapy. More accurate localization of ectopic beats could help guide therapy, thereby reducing procedural time and improving success rates.

### Tuning the bases improves results

One interesting feature of PBR is that its bases can be tuned to a specific clinical question. From a 12-lead ECG, the LV or RV origin of an ectopic beat can often be determined without issues. In this study we have shown that, using a basis specific to the LV or RV, a higher accuracy of electrogram reconstruction is achieved, along with a reduction in origin–localization outliers. One could argue that a more localized basis might be even more beneficial. However, according to a study investigating the accuracy of human interpretation of 12-lead ECG, an experienced cardiologist can correctly identify the ventricle of origin (LV vs RV) in 76.6% of the cases based on the 12-lead ECG, but further sublocalization within the ventricles was accurate in only 38.1%. Thus, one should be careful limiting the basis to very specific regions, as this increases the risk of selecting a basis that is unsuitable for reconstruction of that beat. [7]

Interestingly, the AP model used did not affect reconstruction quality, and a simplified model of AP propagation was found to perform as well as a fine-grained monodomain simulation. This may be explained by the fact that the truncated basis elements reflect the *spatial* potential patterns, mainly reflecting the order of activation. Consequently, as demonstrated in Online Fig. 1, the basis patterns obtained with SVD using different models are very similar, unless spatial differences exist such as limited regions of beat origins.

This means that PBR leaves temporal aspects largely unconstrained and it is unlikely that incorporating *temporal* patient-specific characteristics will benefit the results. However, including *spatial* information such as infarcted regions could directly influence reconstruction quality: These characteristics will have a large impact on simulated beat propagation and will therefore be incorporated in the truncated spatial basis. Similarly, incorporating *spatiotemporal* information such as abnormal regional changes to the AP may lead to improved reconstructions. These results suggest that even simpler methods for simulating propagating waves, for example using the eikonal equations, may be applicable to PBR in its basic form. However, such methods do not offer as clear a road to integrating patient-specific spatiotemporal characteristics such as local changes in the ionic balances. As the dogs used in this study had normal hearts, we were not able to investigate these hypotheses.

### Parameter dependency

PBR is able to reconstruct electrophysiologically relevant patterns using a limited number of basis elements (typically 9) generated from only a few simulated beats (6 or more). This is possible, because a linear combination of these basis elements still spans a large enough solution space, even when this truncated basis only consists of the first, simple basis patterns. The Lasso method allows any linear combination, but gives preference to sparse solutions, thereby reducing the influence of ill-posedness. The combination of a truncated realistic basis with Lasso optimization will result in solutions based on a small number of electrophysiologically relevant ‘building blocks.’

An open question is how to automatically determine the number of elements needed in the truncated basis. With too few elements, the basis will not span the full range of possible potential patterns and the method will be unable to reconstruct all physiologically relevant cases. If too many elements are used, the ill-posedness of the inverse problem will dominate and allow noise-like influence of the ‘less important’ basis elements to obscure the real solution. In this study, we found that the optimal basis size was close to 10. For clinical applications, where no invasively measured potentials are available, methods are needed that automatically determine the optimal basis size. These should aim to find a balance where ill-posedness is reduced without overly constraining the solution space.

We have not investigated the dependency of PBR on the number of body-surface electrodes. Earlier work by our group shows that ECGI in general needs at least 80 electrodes on the body surface for accurate reconstruction of heart-surface potentials [4]. However, PBR’s lower dimensionality due to the sparse basis might warrant a lower number of body-surface electrodes. Especially in a clinical setting, where the 12-lead ECG is a commonly used tool, its reduced set of only 9 electrodes would make a practical alternative. Future studies should investigate whether this low number of electrodes in combination with PBR does indeed provide accurate results.

### Limitations and future work

One important limitation of our current PBR implementation is that the basis is created from cellular AP simulations, whereas the actual inverse reconstruction is in terms of local electrograms. The cellular AP reflects potential differences over the cell membrane, whereas local electrograms reflect extracellular potential differences over larger regions of tissue. It is possible PBR results could be improved by using simulated local electrograms instead of cellular APs.

In the validation part of this study, we were limited by the healthy status of the animals and could not investigate the effect of (local) tissue abnormalities (e.g., myocardial infarction). We had no information about anisotropy of the tissue, so this was not included in the simulations. However, we expect the more complicated spatial patterns resulting from anisotropy can already be reconstructed by combining our current basis vectors. Future work may be needed to show if this is true.

### Other approaches

We have presented a method to include electrophysiological data in the potential-based formulation of the inverse problem, but this was not the first attempt to achieve this. For example, He et al. [12] proposed to solve the forward problem based on potentials from an anistropic heart model and then compare the resulting body-surface potentials to recordings. By optimizing the computed body-surface potentials, they obtained an estimate for the heart-surface potentials. Wang et al. [30] employed a statistical framework to constrain inverse solutions to realistic transmembrane potential dynamics.

In a study by van Oosterom [27], a method was presented to reconstruct the heart-surface potentials based on a priori knowledge about their spatial covariance. This knowledge is usually not available, and this method therefore cannot be applied in practice. However, with methods similar to those presented in the present paper, knowledge about the spatial covariance could be simulated, making it worthwhile to re-investigate this approach.

In a recent study by Lopez-Rincon et al. [13], heart-surface potentials were reconstructed with the help of a simulation based on the bidomain equations, similar to the TNNPb monodomain simulations performed in this manuscript. We have shown that, in our implementation, such a detailed cellular implementation does not contribute to the accuracy of reconstructions.

Some implementations of the inverse problem of electrocardiography are not based on a potential-based formulation, as in this study, but define cardiac activity in terms of activation wavefronts or other equivalent sources. In the wavefront-based formulation of the inverse problem (cellular), electrophysiology models have always been part of the reconstruction process, e.g., by including a model of the expected transmembrane potentials [9, 26] or modeling the activation wavefront as a physiologically inspired propagating curve [10].

Although our method was not the first to include electrophysiological data to improve the inverse reconstruction of electrical activity on the heart, to the best of our knowledge it is the first to apply it to obtain both activation and recovery patterns and validate these in vivo.

## Conclusion

We have introduced and validated PBR, a novel method to noninvasively reconstruct epicardial potentials from body-surface potentials. By incorporating simulated electrophysiological input in the regularization of the inverse problem of electrocardiography, more information is recovered in the reconstructed epicardial electrograms. Reconstruction of recovery time, in particular, is improved with this method. While we found that the level of temporal detail in the simulations did not affect the results, the inclusion of spatial characteristics (i.e., suspected LV or RV origin) did improve accuracy. Noninvasive imaging of recovery abnormalities with PBR can greatly benefit risk assessment and improve understanding of their role in arrhythmias. This may help to answer clinical questions with improved accuracy.

## Electronic supplementary material

Below is the link to the electronic supplementary material.

Online Figure 1 Bases created with PBR for different AP models and simulated beat origins, in one dog. Each row shows bases elements 2–10 as created with a specific AP models (FHN, LR92, TNNPa or TNNPb) and a specific region of simulated origins (full epicardial surface [`all'], only the LV [`left'], or only the RV [`right']). The elements are shown in reducing order (left-to-right) of contribution to the simulated potentials. Regardless of AP method and beat origins, the first basis elements capture simple patterns and later elements capture more complex patterns. There is a clear difference between basis elements of different beat origins (all vs left vs right), but less difference between basis elements of different AP models. (pdf 3886 KB)
