# Supplementary figures and images for: Physiology-based regularization of the electrocardiographic inverse problem
Source: Med Biol Eng Comput. 2016 Nov 21;55(8):1353–65. doi: 10.1007/s11517-016-1595-5 (PMC5544815; doi:10.1007/s11517-016-1595-5)

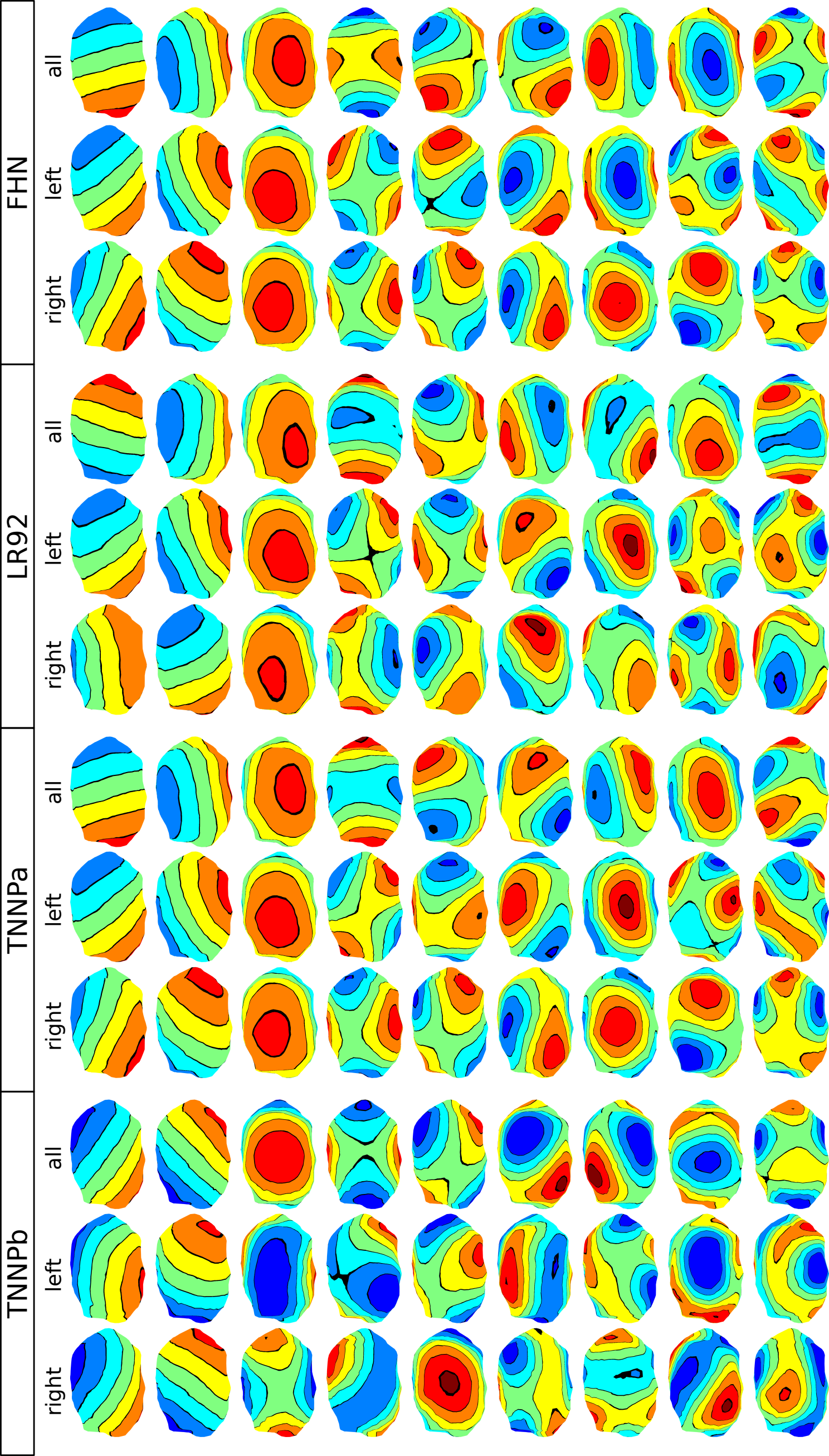

Supplement: Supplementary file 1 — Bases created with PBR for different AP models and simulated beat origins, in one dog. Each row shows bases elements 2–10 as created with a specific AP models (FHN, LR92, TNNPa or TNNPb) and a specific region of simulated origins (full epicardial surface [`all'], only the LV [`left'], or only the RV [`right']). The elements are shown in reducing order (left-to-right) of contribution to the simulated potentials. Regardless of AP method and beat origins, the first basis elements capture simple patterns and later elements capture more complex patterns. There is a clear difference between basis elements of different beat origins (all vs left vs right), but less difference between basis elements of different AP models. (pdf 3886 KB) [file 11517_2016_1595_MOESM1_ESM.pdf]
